# Do endometrial lesions require removal? A retrospective study

**DOI:** 10.1186/s12905-019-0756-8

**Published:** 2019-05-06

**Authors:** Ting Jiang, Qing Yuan, Qin Zhou, Yiping Zhu, Siji Lv, Yanling Cao, Qin Wang, Kunming Li, Dong Zhao

**Affiliations:** 1 Department of Gynecology, Shanghai First Maternity and Infant Hospital, Tongji University School of Medicine, #2699 West Gaoke Road, Pudong New District, Shanghai, 200040 People’s Republic of China; 2 Department of Gynecology, Obstetrics and Gynaecology Hospital of Fudan University, #419 of Fangxie Road, Huangpu District, Shanghai, 200011 People’s Republic of China; 3 Department of Biostatistics, School of Public Health Fudan University, 138# Yixueyuan Road, Shanghai, 200032 People’s Republic of China; 4 Department of Gynecology and Obstetrics, Punan Hospital of Shanghai, #219 of Linyi Road, Pudong New District, Shanghai, 200125 People’s Republic of China; 5 Center of Reproductive Medicine, Shanghai First Maternity and Infant Hospital, Tongji University School of Medicine, #2699 of Gaoke Road (west), Pudong New District, Shanghai, 200040 People’s Republic of China; 6 Department of Obstetrics and Gynecology, Shanghai Ninth People’s Hospital, Shanghai Jiao Tong University School of Medicine, #639 Zhi Zao Ju Road, Huangpu District, Shanghai, 200011 People’s Republic of China

**Keywords:** Asymptomatic, Pre-menopausal, Post-menopausal, Hysteroscopy, Risk factors

## Abstract

**Background:**

This study aimed to evaluate the management of asymptomatic intrauterine lesions detected by ultrasonography.

**Methods:**

Patients who underwent diagnostic hysteroscopy for asymptomatic lesions, including pre- and post-menopausal endometrial polyps, post-menopausal endometrial thickening (ET ≥5 mm) and reduplicative endometrial heterogeneity detected by transvaginal ultrasonography (TVUS), were recruited for this study.

**Results:**

In the 792 recruited patients, the symptom-free focal masses within the uterine cavity detected by TVUS included 558 patients with pre- or post-menopausal endometrial polyps and 234 patients with postmenopausal endometrial thickening. No pre-menopausal patient presented with carcinoma. The polyp diameter (PD) was not identified as an independent risk factor for malignancy in this study. A significant difference (*P* = 0.036, < 0.05) in both benign and malignant endometrial lesions was observed between two groups of post-menopausal women stratified using an endometrial thickness cut-off of ≥11 mm.

The TVUS was highly sensitive (94%) for pre-menopausal polyps. This technique had a specificity and positive predictive value of 84.4 and 92.7%, respectively, for postmenopausal polyps. The TVUS was clearly valuable for ruling out polyps, as indicated by a negative likelihood ratio (LR-) of 0.087.

Among postmenopausal women with endometrial thickening, the area under the receiver operating characteristic curve was 0.828 (*P* < 0.001). An ET cut-off value of 12.5 mm yielded a sensitivity of 72.7% and specificity of 86%.

**Conclusion:**

We recommend follow-up alone for women with asymptomatic uterine polyps, particularly those who are pre-menopausal. Additionally, gynaecologists should consider risk factors such as age, obesity, polycystic ovarian syndrome, and diabetes. Prospective long-term follow-up studies should be conducted after hysteroscopic polypectomy to evaluate the recurrence rate of endometrial lesions.

## Background

Endometrial carcinoma is a common type of gynaecological malignancy. Hysteroscopic investigation, rather than dilatation and curettage is a widely accepted diagnostic method for of endometrial cancer. In a recent report, only one of 35 asymptomatic post-menopausal women with an endometrial thickness exceeding 4 mm presented with adenocarcinoma [1]. In a study of 268 women undergoing diagnostic hysteroscopy, Giannella reported only four cases of endometrial cancer and two cases of atypical endometrial hyperplasia [2]. Both studies reported a considerably low incidence of premalignant or malignant lesions in asymptomatic post-menopausal women. In another words, many of these women may have undergone unnecessary surgeries. Furthermore, the guidelines for asymptomatic endometrial lesions appear to be somewhat ambiguous.

According to the 2010 guideline for Asymptomatic Endometrial Thickening by the Society of Obstetricians and Gynaecologists of Canada (SOGC) [3] if a patient presents with an endometrial thickness > 11 mm or any other positive ultrasonographic findings, such as increased vascularity, an inhomogeneous endometrium, or particulate fluid, further investigations should be considered after accounting for various factors, such as age, obesity, diabetes, hypertension, and late menopausal age.

According to the 2012 Practice Guidelines for the Diagnosis and Management of Endometrial Polyps by the American Association of Gynaecologic Laparoscopists (AAGL) [4], conservative management is reasonable, particularly for asymptomatic small polyps. By contrast, removal for pathological assessment is only indicated for symptomatic polyps. In a study included 112 women with endometrial polyps, which were expectantly managed over a median period of 22.5 months (range, 6–136). There was no association between women’s demographic characteristics or polyps’ morphology and their growth rates. Spontaneous regression appeared to occur more frequently in premenopausal women (*P* = 0.016) and in those who presented with abnormal uterine bleeding at diagnosis (*P* = 0.004); however, the differences did not reach statistical significance after correction for multiple comparisons. [5]

In China, a national guideline for asymptomatic endometrial lesions has long been needed. Currently, different institutions and even different physicians at the same institution might apply different criteria when determining whether to intervene surgically in a case of postmenopausal endometrial thickening (ET). An existing guideline for abnormal uterine bleeding (AUB) indicated that asymptomatic polyps with a diameter < 1 cm have a regression rate of 27%. Patients with larger polyps, symptomatic polyps or infertility should undergo hysteroscopic polypectomy [6].

Once a susceptible endometrial polyp or a post-menopausal endometrial thickness > 5 mm has been identified by transvaginal ultrasonography (TVUS), the patient will likely be subjected to hysteroscopic polypectomy and/or curettage. Patients with an endometrium that remains inhomogeneous over time might also undergo surgical investigation after a period of observation. This preference for aggressive treatment might be partially attributable to the characteristically strict doctor–patient relationship in China.

To our knowledge, there is little evidence to indicate the appropriate interventions of asymptomatic intrauterine lesions. We therefore aimed to investigate this dilemma and identify a feasible standard for intervention in the Chinese population.

## Methods

Patients who presented with asymptomatic lesions at Shanghai First Maternity and Infant Hospital from June 2013 to June 2015 were recruited. The study protocol was approved by the Ethical Committee of the Tongji University School of Medicine.

In addition to the medical history, each patient’s endometrial thickness (ET), polyp diameter (PD) and pathological diagnosis was documented to evaluate the necessity of surgery and validity of the practice.

All subjects underwent hysteroscopy, which remains the gold-standard diagnostic technique for endometrial lesions [3]. Asymptomatic lesions included pre- or post-menopausal endometrial polyps, post-menopausal endometrial thickening (endometrial thickness ≥ 5 mm) or reduplicative endometrial heterogeneity detected by TVUS. Inclusion criteria: Premenopausal endometrial insufficiency or uterine cavity occupying. On the 3-5th day of menstruation next month, review the ultrasound if there is still uneven endometrium or uterine cavity lesion; Postmenopausal endometrium ≥5 mm or uterine cavity lesion. Exclusion criteria: The growth stage patients with thicker endometrium were followed up by the clinic. Hysteroscopic treatment was required if there was abnormal uterine bleeding. And all the hysteroscopic surgery for abnormal uterine bleeding is beyond our scope of discussion.

Results were expressed as means±standard deviation (SD) or standard error of mean (SEM). Data were analysed by SPSS 22.0 (Chicago, IL, USA). The comparison between the two groups was performed with the chi-square test and *P* < 0.05 indicates statistically significance. A multi-logistic regression analysis was applied to study the risk factors for malignancy in patients with asymptomatic endometrial lesions.

The study protocol was approved by the Ethical Committee of the Tongji University School of Medicine.

## Statistics

All data were analysed using SPSS 19.0 (SPSS, Inc., Chicago, IL, USA). The chi-square test was applied to comparisons between groups, and a *P* value < 0.05 was considered to indicate a statistically significant difference.

## Results

A total of 792 patients with symptom-free focal masses within the uterine cavity detected by TVUS were recruited; these included 558 patients with pre- or post-menopausal endometrial polyps and 234 patients with post-menopausal endometrial thickening (endometrial thickness ≥ 5 mm) who underwent hysteroscopic resection and diagnostic curettage.

The ages of women with asymptomatic polyps ranged from 21 to 57 years in the pre-menopausal group and from 40 to 78 years in the post-menopausal group, with respective mean ages of 38.42 ± 7.60 and 60.85 ± 5.41 years, respectively. In the asymptomatic endometrial thickening group, the women’s ages ranged from 46 to 81 years, with a mean of 60.94 ± 5.88 years (Table 1).

**Table 1:** The ages of the patients of asymptomatic endometrial lesions

	Ages (years)		
	Maximum	Minimum	Mean ± SD
Asymptomatic polyps			
Premenopausal (355)	21	57	38.42 ± 7.60
^a^Postmenopausal (202)	40	78	60.85 ± 5.41
Asymptomatic endometrial thickening			
Postmenopausal (234)	46	81	60.94 ± 5.88

^a^missing value: *n* = 1

Asymptomatic endometrial lesions were associated with a morbidity rate of 86.9%, as summarised in Table 2. Of these cases, 13.1% were normal, 72.6% had polyps (405/558), 9.1% had fibroids/adenomyomas (51/558), 1.4% had simple/complex hyperplasia (8/558), 0.5% had atypical hyperplasia (3/558) and 1.5% had carcinoma (10/558). No carcinomas were observed in the pre-menopausal group, and only two patients in that group had atypical hyperplasia. The pre- and post-menopausal groups differed significantly in the incidence of both benign and malignant endometrial lesions (*P* = 0.000, < 0.05, respectively).

**Table 2:** Final diagnosis in the pre−/post-menopausal of asymptomatic intrauterine polyp

	Premenopausal (*N* = 355)		Postmenopausal (*N* = 203)		Total (*N* = 558)	
	N	%	N	%	N	%
Proliferative/secretory/atrophy	58	16.3	15	7.4	73	13.1
Polyp	262	73.8	143	70.4	405	72.6
Fibroid	24	6.8	27	13.3	51	9.1
Hyperplasia without atypical	6	1.7	2	1.0	8	1.4
Atypical hyperplasia	2	0.6	1	0.5	3	0.5
Carcinoma	0	0	10	4.9	10	1.8
Other	3	0.9	5	2.5	8	1.5

In the pre-menopausal group, 14.9% (29/195) of patients with a PD ≥10 mm on TVUS were normal (Table 3). Only one case of atypical hyperplasia (0.5%, 1/198) was observed among patients with a PD ≥10 mm, and the incidence of this lesion type was 0.6% (2/363), regardless of menstrual status. There was no statistical difference between the premenopausal and postmenopausal groups (*P* = 1.0, > 0.05).

**Table 3:** The diagnosis of the polyp in pre−/post-menopausal groups divided by diameter

	Premenopausal						Postmenopausal					
	Diameter > = 10 mm (*N* = 195)		Diameter < 10 mm (*N* = 150)		Total (*N* = 363)		Diameter > = 10 mm (*N* = 81)		Diameter < 10 mm (*N* = 113)		Total (*N* = 203)	
	N	%	N	%	N	%	N	%	N	%	N	%
Proliferative/secret-ory/atrophy	29	14.9	26	17.3	58	16.3	2	2.5	13	11.5	15	7.4
Polyp	144	73.8	114	76	262	73.8	52	64.2	84	74.3	143	70.4
Fibroid	18	9.2	3	2.0	24	6.8	14	17.3	11	9.7	27	13.3
Hyperplasia without atypical	2	1.0	4	2.7	6	1.7	2	2.5	0	0	2	1.0
Atypical hyperplasia	1	0.5	1	0.7	2	0.6	0	0	1	0.9	1	0.5
Carcinoma	0	0	0	0	0	0	8	9.9	2	1.8	10	4.9
Other	1	0.5	2	1.4	3	0.9	3	3.7	2	1.8	5	2.5

Furthermore, 9.9% (8/81) of post-menopausal patients with a PD ≥10 mm had carcinoma; among all post-menopausal subjects, the incidence rates of endometrial carcinoma and atypical hyperplasia were 4.9% (10/203) and 0.5% (1/203), respectively. A chi-square test revealed no significant difference in these incidence rates between the groups divided by PD (*P* = 0.055, > 0.05) (Table 3).

Asymptomatic post-menopausal subjects were divided into two groups according to endometrial thickness (Table 4). Significant differences in the incidence rates of benign and malignant endometrial lesions were observed between the two groups (*P* = 0.002, < 0.05, respectively). The vast majority of lesions were benign, including 98.2% in the group with ET from 5 to 10 mm, and 87.5% in the group with ET ≥11 mm. The malignancy rates in those two groups were 1.2% (2/172) and 7.8% (5/64) respectively.

**Table 4:** The diagnosis of asymptomatic endometrial thickening divided by ET

	ET > =5 mm (*N* = 234)		ET > =11 mm (*N* = 62)	
	N	%	N	%
Proliferative/secretory/atrophy	71	30.3	11	17.7
Polyp	134	57.3	39	62.9
Fibroid	12	5.1	2	3.2
Hyperplasia without atypical	3	1.3	1	1.6
Atypical hyperplasia	4	1.7	3	4.8
Carcinoma	7	3.0	5	8.1
Other	3	1.2	1	1.6

A multi-logistic regression analysis was applied to study the risk factors for malignancy in patients with asymptomatic endometrial lesions. The results revealed that age and ET were correlated with malignancy, whereas other factors such as BMI, obesity, diabetes and hypertension were not correlated with malignancy.

**Fig. 1:**
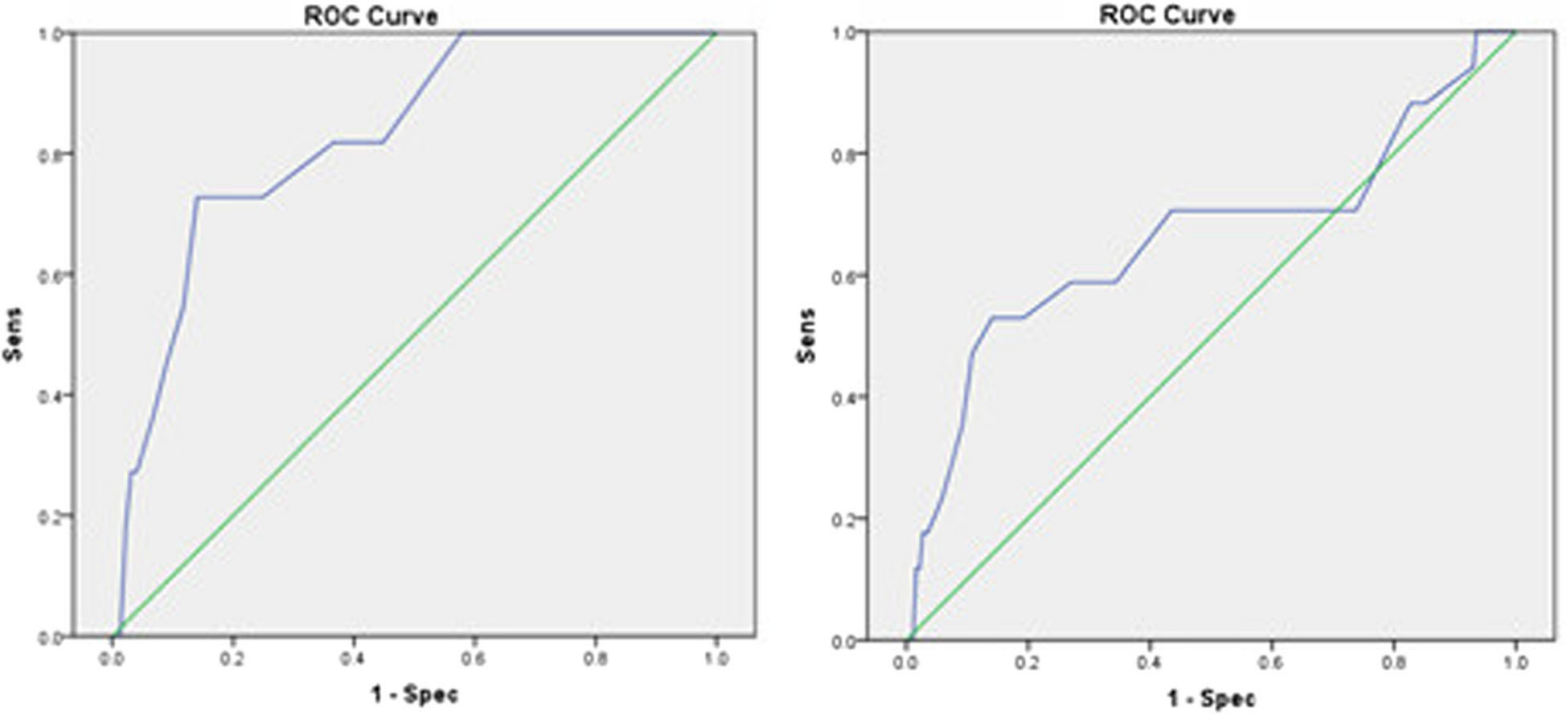
Receiver operating characteristic (ROC) curve of endometrial thickness (ET) for the assessment of endometrial carcinoma. At left, post-menopausal women with endometrial thickening (*N* = 234, two missing values): the area under the ROC curve (AUC) was 0.828 (*P* < 0.001). An ET cut-off value yielded a sensitivity of 72.7% and specificity of 86%. At right, post-menopausal women with endometrial thickening, regardless of polyp status (*N* = 436, 49 missing values): the AUC was 0.662 (*P* = 0.024, < 0.05). An ET cut-off value of 10.5 mm yielded a sensitivity of 52.9% and specificity of 80.8%

A receiver operating characteristic curve was used to assess the prediction value of ET for endometrial carcinoma. The sensitivity was 72.7% if an ET value of 12.5 mm was set as a cut-off, and the specificity was 86% (left part of Fig. 1). All above data corresponded to post-menopausal women with endometrial thickening. When we considered the ET of post-menopausal women regardless of PD, the area under the curve (AUC) was 0.662 (*P* = 0.024, < 0.05), as shown in the right part of Fig. 1. An ET cut-off value of 10.5 mm yielded a sensitivity of 52.9% and specificity of 80.8%.

**Fig. 2:**
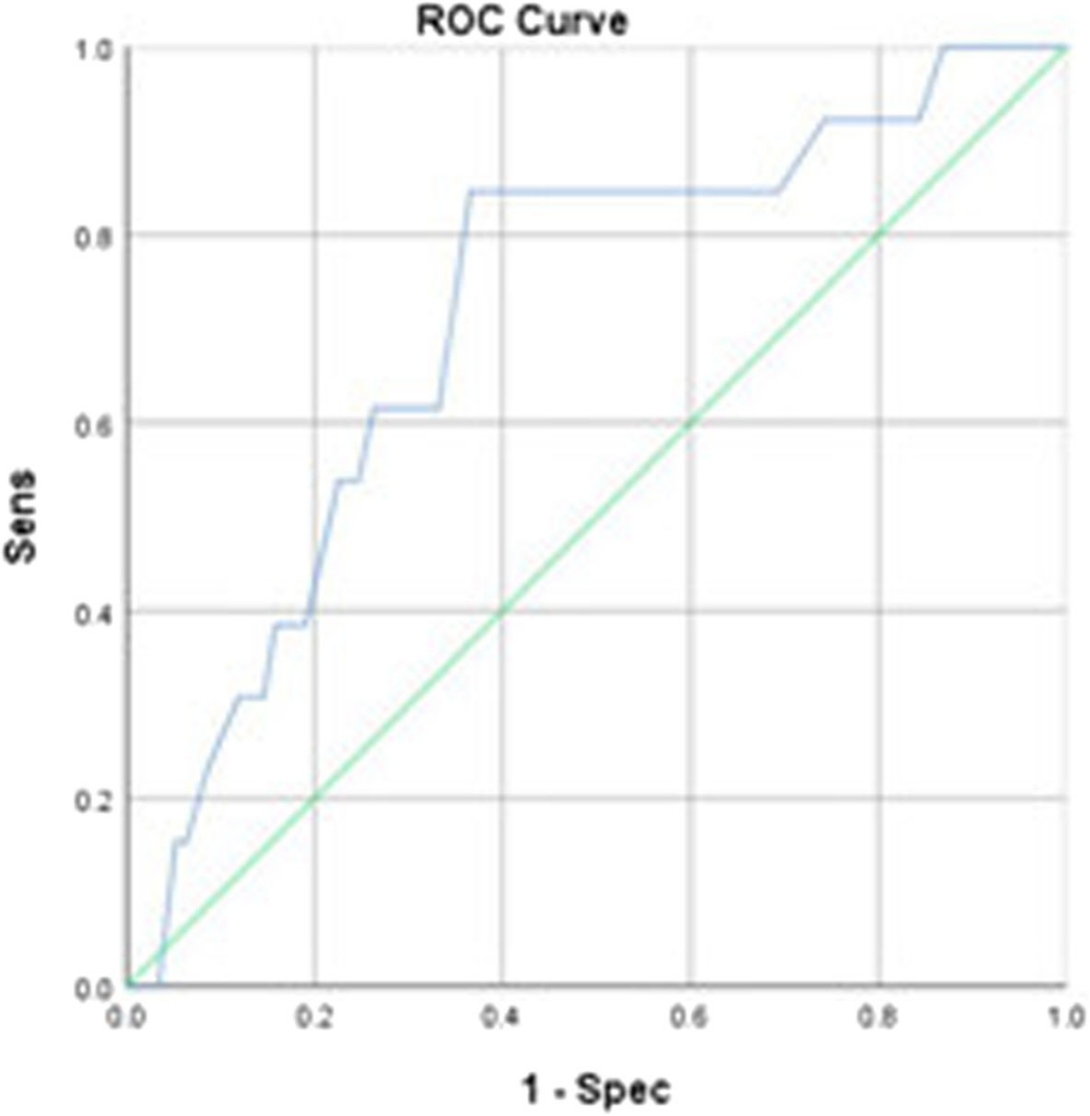
The use of age to predict malignancy yielded an area under the receiver operating characteristic curve of 0.715 (*P* = 0.008). An age cut-off value of 48.5 years yielded a sensitivity of 84.6% and specificity of 42.2%

An AUC of 0.715 (*P* = 0.008) was determined when using age to predict malignancy. An age cut-off value of 48.5 years yielded a sensitivity of 84.6% and specificity of 42.2% (Fig. 2).

Patients with an inhomogeneous endometrium detected by TVUS were divided into several groups according to the presence or absence of a polyp by hysteroscopic investigation (Table 5). Regardless of the menopausal status, these cases were usually identified as endometrial polyps, with prevalence rates of 67.3% (35/52) and 81.48% (66/81) in the pre- and post-menopausal groups, respectively, and 76.76% (109/142) overall.

**Table 5:** Inhomogenous endometrial showed by ultrasonography, which divided by whether polyp or polyp-free found by hysteroscopy in the uterine cavity

	Premenopausal (*N* = 52)				Postmenopausal (*N* = 81)				Total (*N* = 142)			
	Polyp (*N* = 35)		Free-polyp (*N* = 17)		Polyp (*N* = 66)		Free-polyp (*N* = 15)		Polyp (*N* = 109)		Free-polyp (*N* = 33)	
	N	%	N	%	N	%	N	%	N	%	N	%
Proliferative/secretory/atrophy	9	25.7	11	64.7	2	3.0	10	66.7	13	11.9	22	66.7
Polyp	21	60.0	2	11.8	54	81.8	2	13.3	80	73.4	4	12.1
Fibroid	2	5.7	0	0	6	9.1	0	0	8	7.3	0	0
Hyperplasia without atypical	3	8.6	3	17.7	0	0	2	13.4	4	3.7	5	15.2
Atypical hyperplasia	0	0	0	0	2	3.0	0	0	2	1.8	0	0
Carcinoma	0	0	1	5.9	2	3.0	1	6.7	2	1.8	2	6.1

The TVUS was highly sensitive (94%) for pre-menopausal polyps. For post-menopausal polyps, it yielded a specificity of 84.4% and positive predictive value of 92.7%. TVUS appears to be valuable for ruling out polyps, as indicated by a negative likelihood ratio (LR-) of 0.087.

## Discussion

This study observed an overall incidence of atypical endometrial hyperplasia and carcinoma of 2.3% among asymptomatic patients (Table 2). Additionally, 142 cases of inhomogeneous endometrium were detected by TVUS, with ET values ranging between 2 and 22 mm (9.67 ± 3.88 mm, nine missing values). Four subjects had endometrial polyps detected by hysteroscopy but not TVUS (Table 5).

Table 6 suggests that TVUS might not be a perfect screening modality. The American Cancer Society concluded that there was insufficient evidence to recommend any routine screening (TVUS or endometrial biopsy) for endometrial cancer [7]. Given the low specificities of these modalities, the screening of asymptomatic women would result in unnecessary examinations.

**Table 6:** TVUS for the diagnosis of intrauterine pathology^a^ in pre- and post-menopausal polyps

	Sens %	Spec %	PPV %	NPV %	LR+	LR-
Premenopausal polyps	94.0	68.6	84.0	52.2	2.99	0.087
Postmenopausal polyps	51.8	84.4	92.7	31.4	3.32	0.57

*Sens* sensitivity, *Spec* specificity, *PPV* positive predictive value, *NPV* negative predictive value, *LR*+ positive likelihood ratio, *LR-* negative likelihood ratio

^a^Endometrial polyps, intrauterine fibroids and endometrial cancer

Saline infusion ultrasonography (SIS) can improve the accuracy of uterine lesion diagnosis; this technique has a high specificity and sensitivity of 94 and 88%, respectively, according to a meta-analysis conducted in 2015 [8]. However, this procedure has not been widely adopted as a routine evaluation in China because of its time-consuming nature. Although hysteroscopy is currently considered the gold standard [9], the potential value of SIS should be considered, as this procedure is painless, cost-effective and minimally invasive [10–12]. SIS provides an excellent diagnostic technique to diagnose the size and the anatomic location of endometrial polyps.

Hysteroscopy enables the direct visualisation of the endometrial cavity while simultaneously providing the opportunity to remove lesions such as polyps and submucosal fibroids. A recent systematic review and meta-analysis estimated the overall success rate of this procedure to be 96.9% [13].

Nevertheless, hysteroscopy has a false negative rate of 3%; therefore, the endometrium should be sampled even if the uterine cavity appears to be normal [14]. In addition, hysteroscopy is invasive and expensive compared to SIS [15].

Unnecessary hysteroscopy could be considerably reduced without compromising the sensitivity for detecting malignant disease if the criterion for surgical intervention was set at an ET of ≥12.5 mm (Table 4).

In this study of patients with polyps, 40.7% (227/558) of all subjects and 35.8% (127/355) of pre-menopausal subjects had not been given the option of observation. However, hysteroscopy detected only two patients with atypical hyperplasia in the pre-menopausal group. All biopsies of the 127 pre-menopausal women who underwent hysteroscopy without observation yielded benign results. Our data suggest that immediate hysteroscopy might not be the best option for women with polyps and no fertility issues. Rather, observation could reasonably reduce over-treatment.

A recently published meta-analysis of 10,572 pre- and post-menopausal women reported a prevalence rate of 3.57% for atypical endometrial hyperplasia and carcinoma [16], which is comparable to the findings from our study.

Recurrence rate of EPs after resection is unknown. The significantly increased incidence of colorectal polyps in cohorts that also had EPs might indicate that patients with EPs should be also referred for colonoscopy. EPs have the lowest incidence of malignant transformation as compared to colon, urinary bladder, oropharyngeal, nasal and laryngeal carcinomas [17].

A total of 188 patients were included in a study [18]. The most common histopathological results were endometrial polyp, atrophic endometrium, and surface epithelium (26.6, 22.3, and 12.8%, respectively). None of the 57 patients without vaginal bleeding had endometrial cancer. This conclusion is consistent with our research. But it was not determine the cut-off value. And it point that the presence of vaginal bleeding was significantly associated with the diagnosis of endometrial cancer and any endometrial disorder (*p* = 0.001 and *p* = 0.000, respectively).

Our research report no significant difference in these incidence rates between the groups divided by PD (*P* = 0.055, > 0.05) in post-menopausal patients’ endometrial carcinoma and atypical hyperplasia. Since the *P* value is close to 0.05, the post-menopausal patient with PD ≥10 mm should undergo the operation in our clinical recommendation.

Our analysis suggested that ET and age were risk factors for endometrial malignancy. Among post-menopausal women, an ET cut-off value of 12.5 mm was determined. Our data suggest that PD does not correlate with malignancy. Although ET was identified as a main factor associated with malignancy, the effectiveness of this assessment appeared to decrease. Certainly, morbidity would increase with aging. However, an age cut-off of 48.5 years could not effectively predict malignant disease in asymptomatic women.

The true incidence of asymptomatic polyps remains to be clarified [19–21]. In a study of 1870 subjects, 653 patients had been diagnosed with polyps, 117 were asymptomatic and none of the latter had carcinoma [22]. Another study of 1155 women reported that only one case of endometrial carcinoma was observed among patients with asymptomatic polyps and concluded that hysteroscopic polypectomy could be performed except in cases of polyps with large diameters [23].

The prevalence of polyp among women who underwent diagnostic hysteroscopy and blind polypectomy was more common in the age group of 40-49 years. Polyps manifested as AUB in 45.6% of our study population. The mean size of the polyp was not significantly different between premenopausal and postmenopausal women and single and multiple polyps. Histopathological study of the polyp showed two malignant polyps in our study population. Premalignant lesions i.e., endometrial hyperplasia without atypia and with atypia was found in 33 women. There was one uterine perforation, one cervical tear; one false passage and one patient had mild bleeding after the procedure. In our study, in the mean follow-up period of 37.57 ± 28.12 months, 3.9% (7 women) had recurrence. In the follow-up period of 16.56 ± 18.96 months, 78.9% women didn’t have recurrence [24]. It is comparable to the findings from our study.

It remains uncertain whether the PD of an asymptomatic intrauterine lesion is a critical factor in the surgical indication. In this study, we did not find that the PD was an independent risk factor for malignancy in either pre- or post-menopausal women.

## Conclusion

Asymptomatic polyps and ET are common gynaecological conditions. However, unnecessary interventions are administered in practice. Therefore, the elimination of over-treatment would be ideal. We conclude that asymptomatic patients with polyps, particularly those who are pre-menopausal, likely do not require immediate intervention.

Criteria for surgical intervention, such as an ET ≥12.5 mm could potentially reduce the risk of over-treatment. In asymptomatic young woman with small EPs < 10 mm in size, conservative management can be safely followed by monitoring the polyp growth. EPs located at the fundal and tubocornual regions mechanically affect fertility and disturb normal cellular function due to chronic inflammation. In cases where EPs are a cause of subfertility mechanical hysteroscopic resection is advisable.

EP Detection in either peri- or post-menopausal age, in symptomatic or asymptomatic patients’ calls for meticulous hysteroscopic examination and polypectomy is mandatory. Endometrial curettage is also recommended to rule out sub clinical endometrial hyperplasia or cancer.

An endometrial histopathological examination should be considered, especially for patients with a history of vaginal bleeding.

Prospective long-term follow-up studies after hysteroscopy and polypectomy are needed to evaluate the recurrence rates of endometrial lesions.

## Abbreviations

AUB: Abnormal uterine bleeding
AUC: Area under the curve
ET: Endometrial thickening
PD: Diameters of the polyps
ROC: Receiver operating characteristic
SIS: Saline infusion ultrasonography
TVUS: Transvaginal ultrasonography
